# HF Formation through Dissociative Electron Attachment—A Combined Experimental and Theoretical Study on Pentafluorothiophenol and 2-Fluorothiophenol

**DOI:** 10.3390/ijms23052430

**Published:** 2022-02-23

**Authors:** Maicol Cipriani, Oddur Ingólfsson

**Affiliations:** Department of Chemistry and Science Institute, University of Iceland, Dunhagi 3, 107 Reykjavik, Iceland; mac31@hi.is

**Keywords:** chemoradiation, radiosensitizers, low-energy electron interaction, perfluorination, HF formation, pentafluorothiophenol, 2-fluorothiophenol, dissociative electron attachment

## Abstract

In chemoradiation therapy, dissociative electron attachment (DEA) may play an important role with respect to the efficiency of the radiosensitizers used. The rational tailoring of such radiosensitizers to be more susceptive to DEA may thus offer a path to increase their efficiency. Potentially, this may be achieved by tailoring rearrangement reactions into the DEA process such that these may proceed at low incident electron energies, where DEA is most effective. Favorably altering the orbital structure of the respective molecules through substitution is another path that may be taken to promote dissociation up on electron capture. Here we present a combined experimental and theoretical study on DEA in relation to pentafluorothiophenol (PFTP) and 2-fluorothiophenol (2-FTP). We investigate the thermochemistry and dynamics of neutral HF formation through DEA as means to lower the threshold for dissociation up on electron capture to these compounds, and we explore the influence of perfluorination on their orbital structure. Fragment ion yield curves are presented, and the thermochemical thresholds for the respective DEA processes are computed as well as the minimum energy paths for HF formation up on electron capture and the underlying orbital structure of the respective molecular anions. We show that perfluorination of the aromatic ring in these compounds plays an important role in enabling HF formation by further lowering the threshold for this process and through favorable influence on the orbital structure, such that DEA is promoted. We argue that this approach may offer a path for tailoring new and efficient radiosensitizers.

## 1. Introduction

In recent years, appreciable attention has been paid to the interaction of low-energy electrons (LEEs) with DNA and radiosensitizers applied in cancer therapy [1,2,3,4,5,6]. Although much progress has been made in the fight against cancer, tumor hypoxia still represents an obstacle to traditional cancer therapy. Hypoxia is generally present in solid tumors due to their limited vascularization. The decrease in O_2_ availability in tumor masses may make chemotherapy and radiotherapy ineffective [7,8,9,10]. A more efficient therapy is the concomitant application of radiation with oxygen-mimetic radiosensitizers, most commonly nitro-imidazoles [11,12]. In these electron-affinic radiosensitizers, the nitro group binds with the DNA free radicals generated by ionizing radiation and consequently induces DNA strand breaks [12]. However, at the microscopic level, low-energy electron (LEE) interaction plays an important role in sensitizing cancer cells to radiation [3,13]. The interaction of the ionizing radiation with a biological tissue generates LEEs (<20 eV) with energy distribution that peaks at or below 10 eV, with appreciable contribution close to 0 eV and a tail extending to higher energies [14]. At electron energies below 10 eV, electron-affinic radiosensitizers can be subjected to LEE induced reactions, which yield radical species that can damage DNA. In an aqueous medium, LEEs solvate on a picosecond scale [15]. However, before solvation, an LEE can occupy a vacant virtual orbital of a radiosensitizer, giving rise to a transient negative ion (TNI). If reaction channels are available at these electron energies, a TNI can undergo fragmentation via dissociative electron attachment (DEA), generating a negatively charged ion and neutral counter-fragment(s) [16,17]. This process is in competition with the relaxation of the TNI through autodetachment, i.e., the re-emission of the electron without fragmentation. The electron attachment process, which is the formation of the TNI, is most efficient at low energies, and the autodetachment lifetime decreases rapidly with increasing electron energies. Thus, DEA is most efficient at very low incident electron energies, given that the respective dissociation channel is thermochemically accessible. Hence, exothermic DEA processes, which may proceed close to 0 eV electron energy, are most efficient. The important role of LEEs and DEA in DNA radiolysis was shown by Boudaïffa et al. [1] already in the year 2000, in a study where the authors demonstrated that DEA processes can trigger single and double-strand breaks. This work triggered manifold studies on LEE interaction with DNA components and the fragmentation of negative ions of DNA components, with the bulk of this work being reviewed, for example, in references [18,19,20,21,22]. Furthermore, LEE interaction with radiosensitizers has also been investigated in a number of studies [3,4,5,6,23,24,25]. In these studies, it has, e.g., been shown for 5-halouracils that halogenation increases the DEA cross-sections and such halogenated uracils present sharp peaks with high cross sections in the 0–4 eV electron energy range [23]. In this context, it has also been shown that substitution of thymine with the higher electron affinity 5-halouraci significantly sensitizes DNA to radiation [26]. Similarly, Rackwitz et al. [24] have shown enhanced strand-brake efficiency through DEA to DNA oligonucleotedes when replacing adenine with 2-fluoro adenine, the active component in the chemotherapeutic fludarabine that has also been considered for use in chemoradiation therapy [27,28,29]. Rackwitz et al. [24] associate the observed strand brakes to resonances they observe in gas phase DEA to 2-fluoro adenine at around 5.5 eV and note that these are shifted towards lower energies when compared to DEA to the native adenine. In addition, DEA to the oxygen-mimetic radiosensitizers 2-nitroimidazole and 4(5)-nitroimidazole has been shown to effetely fragment these molecules [5,6].

Dissociative electron attachment is not limited to single bond ruptures but may also involve the rupture of multiple bonds and the formation of new bonds. The generation of new chemical bonds provides additional energy to the system and can thus promote DEA and open new reaction channels otherwise inaccessible at low electron energies. In recent years, dissociative electron attachment (DEA) reactions leading to neutral HF formation from perfluorinated benzene derivatives have been the object of several studies [30,31,32,33]. The formation of HF feeds 5.9 eV into the system, i.e., the bond energy of HF, and can promote reaction channels that involve the rupture of multiple bonds. Ómarsson et al. [30,31] conducted detailed experimental and theoretical investigation of HF formation through DEA to pentafluorotoluene (PFT), pentafluoroaniline (PFA), and pentafluorophenol (PFP). There it was shown that the polarization of the X–H bond plays a determining role in neutral HF formation through the promotion of the formation of an intermediate intramolecular hydrogen bond, X–H···F. In these studies, the authors correlated the different magnitude of the polarization of the X–H bond for X = C, N, and O, with the stabilization of the intermediate X–H···F leading to the HF loss in the respective DEA processes. With respect to the thermochemistry, the HF formation upon DEA to PFP was found to be exothermic, partly attributed the subsequent rearrangement of the charge retaining fragment, while in the case of PFA and PFT, the HF formation was found to be endothermic. In fact, it was also pointed out by Rackwitz et al. [24] that the neutral HF formation may provide the thermochemical prerequisite for the fragmentation effectuated in DEA to 2-fluoro adenine and that such neutral halogen acid formation is frequently observed in DEA to halo-nucleobases.

Motivated by the possibility to promote reaction channels in radiosensitizers through HF formation upon DEA, we extend the previous investigations and compare the two compounds, pentafluorothiophenol (PFTP) and 2-fluorothiophenol (2-FTP). A comparison between PFTP and 2-FTP is informative as both these compounds may form the intermediate X–H···F to a fluorine in the ortho position of the aromatic ring and thus dissociate by neutral HF loss up on electron attachment. However, the perfluorination of PFTP changes the order of the involved molecular orbitals and also favorably influences the thermochemistry of the process. Furthermore, the comparison of PFTP to PFP is interesting because S and O atoms have similar electron configurations, being neighbors in the same group within the periodic table. Because S is less electronegative than N and O, the process involving the HF formation will not be as well supported by the polarization of the S–H bond. However, the orbital structure of S is more extended than that of O (principal quantum number (*n*) = 3 as compared to 2 for oxygen), so the S–H bond is longer and weaker than the O–H bond. Thus, R–SH is a stronger acid than R–OH. For the HF formation through DEA to take place close to 0 eV incident electron energy, where the attachment cross section is highest, the electron affinity of the biradical [M-HF] must compensate the energy difference between the cleavage of the two bonds (M–F and M–H) and the formation of the new H–F bond. In addition, 2-FTP has also been the object of a near ultraviolet photodissociation study in regard to the S–H bond cleavage [34], which in turn is a prerequisite for HF formation. 

Here we present a combined theoretical and experimental study where we use PFTP and 2-FTP as model compounds to explore the potential of substitution to enhance the susceptibility of such compounds towards low energy electrons. We present ion yield curves for all DEA fragments observed from these compounds and we explore the influence of fluorination on the relative energies of the respective low-lying anionic states in conjunction with the thermochemistry and reaction paths leading to fragmentation up on electron capture. Specifically, we focus on HF formation as a potential means to supply additional energy into the DEA channels in order to move the fragmentation threshold close to 0 eV, where the attachment cross sections are highest. In this study, we show that the perfluorination of the molecule is not only important with respect to the attachment cross-section but also plays an important role with respect to the orbital structure and the thermochemistry behind the HF formation. We discuss the nature of the SOMOs involved in the electron attachment processes, calculate the thermochemical thresholds of these processes, and compute the minimum energy paths for HF loss for both compounds. We argue that such molecular functionalization may serve as a basis for the design of more efficient radiosensitizers.

## 2. Results and Discussion

**Figure 1:**
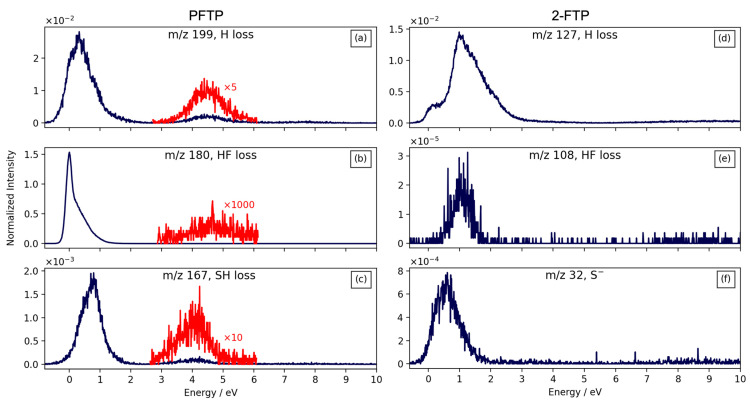
Left: DEA ion yield curves from PFTP for the channels: (**a**) H loss, (**b**) HF loss, and (**c**) SH loss. Right: DEA ion yield curves from 2-FTP for the channels: (**d**) H loss, (**e**) HF loss, (**f**) S^−^ formation. The intensities are normalized with respect to the target gas pressure and the formation of SF_6_^−^ from SF_6_ at 0 eV incident electron energy.

Figure 1 shows negative ion yield curves for observed fragments formed through DEA to PFTP (left) and 2–FBT (right), respectively. The ion yield curves are shown for the incident electron energy range from approximately 0 to 10 eV and are normalized to the respective target gas pressure and the relative cross-section of SF_6_^−^ formation from SF_6_ at 0 eV incident electron energy.

The most pronounced DEA channel for PFTP leads to neutral HF loss from the transient negative ion (TNI) formed in the initial attachment process, that is the formation of [M-HF]^−^. This channel is most significant at threshold, i.e., at 0 eV, but has a higher-lying contribution centered at around 4.7 eV, which is approximately three orders of magnitude lower in intensity. The contribution peaking at approximately 0 eV is distinctly asymmetric towards higher energies, and we anticipate that this is due to overlapping contributions from two distinct resonances. Hydrogen loss is also observed from PFTP at low energies (at approximately 0.3 eV) and through a higher-lying resonance appearing in the ion yield curves at approximately 4.5 eV. The relative, maximum cross-section for the hydrogen loss from this molecule, that is the [M-H]^−^ formation at 0.3 eV, is two orders of magnitude lower than that for the HF formation. However, the relative cross-section for the [M-H]^−^ contribution from PFTP at approximately 4.5 eV is three orders of magnitude higher than that for the [M-HF]^−^ formation at approximately 4.7 eV. This is understandable, as the attachment cross-section is significantly higher close to 0 eV as compared to 0.3 eV; however, at energies significantly above threshold, at approximately 4.5 eV in this case, direct dissociation such as the hydrogen loss is expected to be more efficient as compared to rearrangement processes such as the HF formation. Finally, DEA to PFTP also leads to the loss of SH, i.e., the observation of the anionic fragment [M-SH]^−^. Similar to the HF formation and the H loss ion yield curves, the [M-SH]^−^ ion yield curve has a low energy contribution with an onset at approximately 0 eV and peak intensity at approximately 0.8 eV and a less intense second contribution at higher energy that is centered at approximately 4 eV. The maximum relative cross section for the [M-SH]^−^ is three orders of magnitude lower than that for the [M-HF]^−^ formation, i.e., an order of magnitude lower than that for [M-H]^−^. This channel leads to the formation of the stable pentafluorbenzenide anion. 

Dissociative electron attachment to 2–FBT also leads to the formation of [M-H]^−^ and [M-HF]^−^ through resonances at low incident electron energies. These contributions both have their maxima at approximately 1.0 eV. However, unlike PFTP, the relative cross-section for the HF loss from 2-FTP, i.e., the formation of [M-HF]^−^, is three orders of magnitude lower than that for the direct hydrogen loss, [M-H]^−^. The low energy contribution to the [M-H]^−^ formation from 2-FTP is composed of a contribution at approximately 0 eV, appearing as a low energy shoulder on the main contribution that peaks at approximately 1 eV and is asymmetric towards high energies. We attribute this 0 eV shoulder to ‘hot -band transitions’ or I^−^ (*m*/*z* = 127) from some iodine containing compound residual in the 2-FTP sample or possibly in our inlet system. The assignments of the resonance reflected in the low energy [M-H]^−^ contribution is discussed in more details below. In addition to the [M-H]^−^ and [M-HF]^−^ channels, the formation of S^−^ is also observed in DEA to 2-FTP. This channel is, similarly to the others, most efficient at low energies, with an onset at approximately 0 eV and a maximum cross-section at approximately 0.6 eV. The maximum relative cross-section for the S^−^ formation from 2-FTP is approximately two orders of magnitude lower than that for the hydrogen loss from this compound. 

Hence, while the most pronounced DEA channel for 2-FTP is direct hydrogen loss, HF loss is the dominating DEA channel from PFTP. In fact, the relative cross section for neutral HF formation from 2-FTP is five orders of magnitude lower than that for HF formation from 2-FTP. Furthermore, the onset of the HF loss from 2-FTP is at approximately 0.5 eV, indicating that this channel is endothermic, while the cross section for HF loss from PFTP peaks at approximately 0 eV, as would be expected for an exothermic process. 

**Figure 2:**
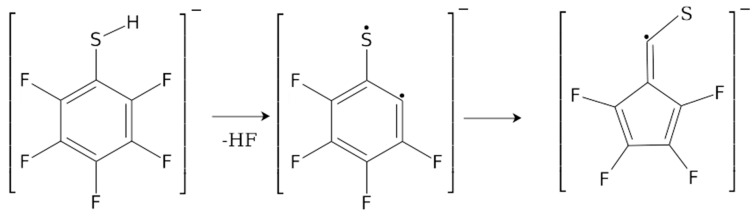
Considered rearrangement of the phenyl ring subsequent to the HF loss upon DEA to PFTP and 2-FTP. In the figure, this is shown for PFTP as an example. In this process, after the HF loss, the 6-membered benzene ring rearranges into a 5-membered ring with exocyclic–CS.

In order to elucidate the thermochemistry and the dynamics of the DEA processes for PFTP and 2-FTP, we have calculated the 0K reaction enthalpies (ΔH_0K_) at the B3LYP D3BJ/aug-cc-pVTZ level of theory for all the observed fragments. These are given in Table 1 along with the thermally corrected values (ΔE_th_) derived by adding the thermal energy correction at room temperature to the parent molecule. This approach is taken as we expect thermal equilibrium for the parent molecules within the inlet system, but not for the DEA fragments formed under single collision conditions. For the HF formation, we additionally considered a rearrangement of the aromatic ring whereby the 6-membered benzene ring is rearranged to a 5-memebered ring with an exocyclic–CS moiety: C_5_F_4_–CS^−^ and C_5_H_4_–CS^−^, respectively, see Figure 2. 

This rearrangement was proposed by Ómarsson et al. [30,31] in their studies on DEA to PFP, PFA, and PFT. Similar to PFTP, effective HF loss from PFP was observed at 0 eV in those studies, while the direct HF loss from PFP, calculated at the B2PLYP/aug-pc-2 level of theory, was found to be endothermic by 0.59 eV. However, in better agreement with the experimental results, a rearrangement leading to a 5-membered ring structure of the anion resulted in a threshold at −0.19 eV. For comparison, we have also calculated the thresholds (ΔE_th_) and 0K reaction enthalpies (ΔH_0K_) for HF formation upon DEA to PFP at the B3LYP D3BJ/aug-cc-pVTZ level of theory and, in fact, at this level of theory, we find the direct HF loss to be exothermic. In addition to the B3LYP D3BJ/aug-cc-pVTZ calculations for PFTP and 2-FTP, shown in Table 1, we have also performed calculations at the ⍵B97X-D3/aug-cc-pVTZ, ⍵B97X-D3/aug-cc-pVQZ, and DLPNO-CCSD(T)/aug-cc-pVQZ levels of theory for HF formation upon DEA to PFTP and PFP; these give qualitatively the same results and are presented as Supporting Material in Table S1. 

According to our calculations (shown in Table 1), the direct HF formation from PFTP is exothermic by 0.29 eV, and rearrangement of the charge-retaining ring only lowers the threshold to −0.35 eV. For the H and SH losses from PFTP, the calculated thermochemical thresholds were found to be 0.13 and 0.32 eV, respectively. This is in good agreement with our experimental results, where the peak intensities for PFTP are found to be at 0.0 eV for the exothermic HF loss, while the endothermic H and the HS losses are shifted to slightly higher energies. Furthermore, the high relative cross-section for the [M-HF]^−^ formation is consistent with the higher attachment cross-section expected at threshold (~0.0 eV) [35].

For 2-FTP, at the B3LYP D3BJ/aug-cc-pVTZ level of theory, we found the thermochemical threshold for the direct HF loss, Table 1, to be 0.42 eV and considering the rearrangement of the ring we found the threshold to be 0.28 eV. The hydrogen loss is found to be energetically less favorable, i.e., endothermic by 0.84 eV. In fact, this is a 0.61 eV higher threshold than the respective threshold for hydrogen loss from PFTP. This is mainly a result of the perfluorination increasing the electron affinity of the charge-retaining fragment C_6_F_4_–S as compared to that for C_6_H_4_F–S. At the B3LYP D3BJ/aug-cc-pVTZ level of theory, we found the 0K adiabatic electron affinities of C_6_F_5_–S and C_6_H_4_F–S to be 2.18 eV and 1.69 eV, respectively.

**Figure 3:**
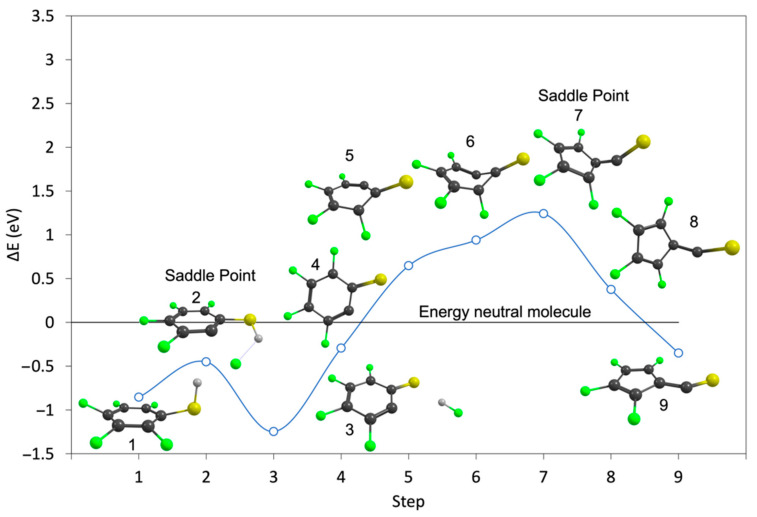
Minimum energy path for the direct HF loss (steps 1–4) and the formation of the 5-membered ring C_5_F_4_–CS^−^ (steps 4–9) from the anionic ground state of PFTP (step 1) calculated using the NEB-TS method at the B3LYP D3BJ/aug-cc-pVTZ level of theory. Step 4 corresponds to the direct HF loss, i.e., without the rearrangement of the ring. The single point energies (open circles) along the reaction paths are relative to the energy of the neutral parent molecule, which is set to 0 eV (horizontal black line). ZPVEs and thermal energy correction for the neutral molecule were taken into account.

**Figure 4:**
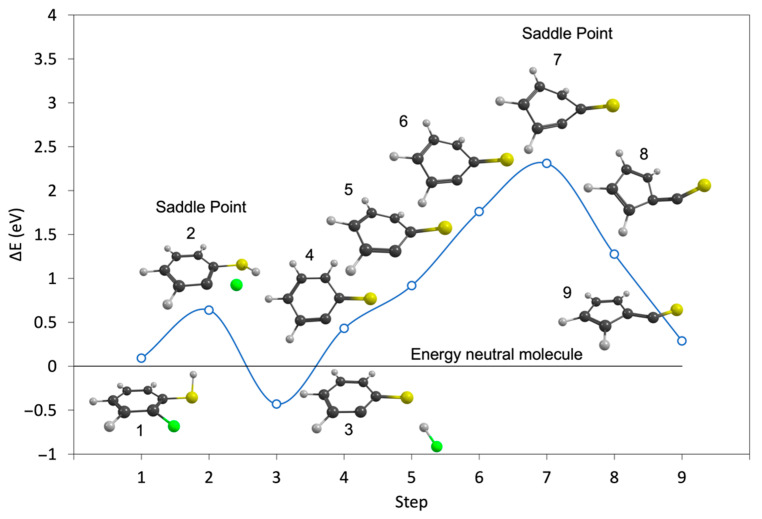
Minimum energy path for the direct HF loss (steps 1–4) and the formation of the 5-membered ring C_5_F_4_–CS^−^ (steps 4–9) from the anionic ground state of 2-FTP (step 1), calculated using the NEB-TS method at the B3LYP D3BJ/aug-cc-pVTZ level of theory. Step 4 corresponds to the direct HF loss, i.e., without the rearrangement of the ring. The single point energies (open circles) along the reaction paths are relative to the energy of the neutral parent molecule, which is set to 0 eV (horizontal black line). ZPVEs and thermal energy correction for the neutral molecule were taken into account.

Despite the fact that the HF loss from 2-FTP is energetically more favorable than the H loss, the H loss dominates the ion yields observed upon DEA to this compound. The significantly higher cross-section for H loss as compared to HF loss must thus be rooted in the dynamics of these processes. To further explore the dynamics of this process, we have performed NEB-TS calculations at the B3LYP D3BJ/aug-cc-pVTZ level of theory to compute the reaction paths for the HF loss from both PFTP and 2–FBT, including the potential rearrangement of the charge retaining phenyl ring as discussed here above. Figure 3 and Figure 4 show the calculated minimum energy paths, on the B3LYP potential energy surface (PES) for the HF formation from PFTP and 2-FTP (step 3) and the subsequent rearrangement of the aromatic ring to form C_5_F_4_–CS^−^ and C_5_H_4_–CS^−^(step 9), respectively, from the anionic ground states of PFTP and 2-FTP (step 1). The total energy of the neutral parent molecule, calculated at the B3LYP D3BJ/aug-cc-pVTZ level of theory, is set at 0 eV, and the black line extending from the *y*-axis marks the relative energy of the neutral ground states. The open circles correspond to the calculated single point energies of the system along the reaction paths, but the blue line is only meant to guide the eye.

Similar to what appears in the minimum energy path for the HF formation from the ground state of the PFP anion, calculated by Ómarsson et al. [31], the formation of the HF, hydrogen-bonded intermediate in step 3 is favored over the molecular anion. Both in the case of PFTP and 2-FTP, see step 2 in Figure 3 and Figure 4, this process (from step 1 to 3) proceeds with an energy barrier. For PFTP it is approximately 0.4 eV and for 2-FTP it is approximately 0.55 eV, relative to the single point energies of the respective relaxed anionic ground states. However, the relaxed PFTP anionic ground state is already 0.85 eV below the respective relaxed neutral ground state of PFTP. The barrier in step 2 and the relaxed C_6_F_4_S^−^ anion, shown in step 4, are thus 0.45 eV and 0.29 eV below the relaxed neutral ground state of PFTP, respectively. Hence, with respect to the neutral, this is a barrierless exothermic reaction and may thus proceed at the 0.0 eV incident electron energy. This is what is observed in the experiments. For 2-FTP, on the other hand, the anionic ground state is 0.09 eV above the neutral ground state, and the activation barrier, shown in step 2, and the relaxed C_6_H_4_S^−^ anion, shown in step 4, lies above the neutral ground state. The saddle point for this process, in step 2, is 0.64 eV above the neutral ground state. Hence, with respect to the neutral, this is a barrierless exothermic reaction and may thus proceed at the 0.0 eV incident electron energy. This is what is observed in the experiments. Furthermore, the energy barrier for the HF loss, step 2 in Figure 4, is comparable to the threshold energy for the direct hydrogen loss. The HF loss can thus only proceed at higher energy, i.e., above the threshold, and this in turn favors the faster, direct hydrogen loss, as is observed in the respective ion yields.

From step 4, the minimum energy path was further calculated considering rearrangement of the ring to form C_5_F_4_–CS^−^ and C_5_H_4_–CS^−^ from PFTP and 2-FTP, respectively. The same procedure was applied in Ómarsson et al. [31]. In both PFTP and 2-FTP, the ring rearrangement to the pentagonal structure (from steps 4 to 9) proceeds through a deformation of the ring with a high energy barrier. For PFTP, this reaction is slightly more exothermic than the direct HF loss; however, as can be seen in Figure 3, there is a 1.24 eV reaction barrier on this path for PFTP (from step 4 to 9). This shows that the HF formation from PFTP at 0 eV threshold energy is direct and proceeds without rearrangement of the aromatic ring. Similarly, we find a reaction barrier of approximately 2.3 eV on this reaction path for 2-FTP, showing that the low energy contribution in the [M-HF]^−^ ion yield from 2-FTP must also be attributed to direct HF loss. 

Similarly, our threshold calculations for the [M-HF]^−^ from PFP, at the B3LYP D3BJ/aug-cc-pVTZ level of theory show that the direct HF loss is also exothermic here (−0.33 eV) and on the minimum energy path for the ring rearrangement in this molecule, Ómarsson et al. [31] found the rearrangement barrier to be close to 2 eV. Therefore, it is reasonable to assume that the [M-HF]^−^ formation from PFP observed at the 0 eV threshold also occurs as a direct process without involving the rearrangement of the ring. 

It is clear from the experiments and the calculations presented above that the perfluorination in PFTP makes the HF loss in DEA energetically more favorable as compared to 2-FTP. Hence, fluorination may potentially be used to sensitize such molecules with respect to DEA by lowering the thermochemical thresholds for these reactions and thus enabling them to proceed at very low energies where the attachment cross section is highest. 

**Figure 5:**
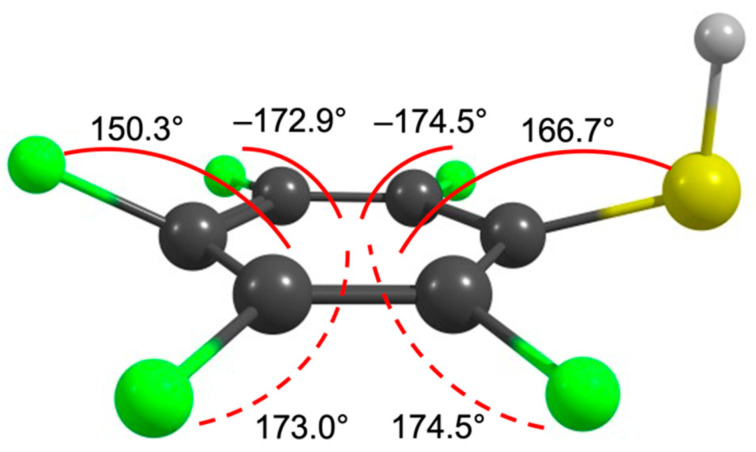
Relaxed geometry of the PFTP anion optimized at the B3LYP D3BJ/aug-cc-pVTZ level of theory. The angles shown in the figure are the angles between the out-of-plane C–F and C–S bonds and the plane of the ring.

With respect to the orbital structure associated with the resonant attachment processes reflected in the ion yields of these compounds, it is worth looking at that of benzene and substituted benzenes. In electron attachment to benzene, the X^2^E_2u_ anionic ground state is formed in the gas phase at 1.15 eV through single electron occupation of the doubly degenerate LUMO e_2u_(π*), as has been assigned through electron transmission spectroscopy [36]. This radical anion distorts due to the Jahn–Teller effect (JT), and the symmetry of the molecule is lowered from D_6h_ to D_2h_, splitting the degenerated e_2u_(π*) LUMO into two components: ^2^A_u_ and ^2^B_u_ [37]. Similarly, the D_6h_ symmetry of the neutral benzene is also broken by substitution at the ring. A single substitution removes the degeneracy of the e_2u_ orbital and lowers the D_6h_ symmetry to C_2v_, whereby the doubly degenerate e_2u_(π*) molecular orbital (MO) splits into the components, a_2_ (π*) and b_1_ (π*) [38,39,40]. At the carbon carrying the substituent, the B_1_-type orbital displays maximum electron density, whilst the A_2_-type has a node at this point. The magnitude of the splitting of these orbitals is influenced by the different combination of the mesomeric and the inductive effect of the respective substituent [41]. While the inductive effect stabilizes both the A_2_ and the B_1_ anion states, the mesomeric effect destabilizes the B_1_ state but generally does not affect the A_2_ significantly. Fluorination of aromatic rings moderately lowers the energy of the π* MOs, but strongly lowers the σ* MOs due to the strong inductive effect of fluorine as compared to its mesomeric effect [42,43]. This is commonly referred to as the perfluoro effect [38,39]. The geometrical structures and the nature of the ground and excited states of fluoro-substituted benzene anions have been studied, both experimentally and theoretically, for example, with electron-spin resonance techniques [44,45] and electron transmission, inner-shell electron energy loss, and magnetic circular dichroism spectroscopy [46] as well as INDO and Hartree Fock calculations [47,48]. Generally, the findings have been that the energy level of the low-lying σ* MO decreases with increasing fluorination and, in the case of C_6_F_6_, the lowest virtual MO is found to be the σ* MO. This is visualized informatively in an energy diagram shown in reference [46]. Similar trends have also been observed in heavily fluorinated pyridine anions [49]. Furthermore, in the theoretical studies [47,48], the authors argued that the structure of the polyfluorinated benzene anions undergoes a distortion due to the pseudo-Jahn–Teller effect (pJT), resulting in a planar carbon structure with C–F bonds out of plane. The extra electron occupies a pseudo-π orbital formed by the mixing of the π* and σ* orbitals. The Q_(b1)_ pJT distortion [47] is given by the vibronic interaction between the totally symmetric σ* state and ^2^B_1_–π state. As may be seen in comparison to the schematic representation in [47], we note that the Q_(b1)_ pJT distortion correlates well with the relaxed structure of the PFTP anion optimized at the B3LYP D3BJ/aug-cc-pVTZ level, shown in Figure 5. 

Both the influence of fluorination on the order of the lowest lying π* and σ* orbitals and the JT distortion up on electron capture is important in DEA to these compounds as a direct dissociation along the substituent’s σ* bond to the aromatic ring is symmetry forbidden from the π* MOs in the C_2v_ point group [50]. Hence, effective coupling between the respective π* and σ* states is required for such dissociation to take place. Occupation of the σ* orbital, on the other hand, can lead to direct dissociation. This may influence the dissociation cross-section significantly, especially where there is strong competition with autodetachment, and the survival probability of the initially formed TNI is determined.

**Figure 6:**
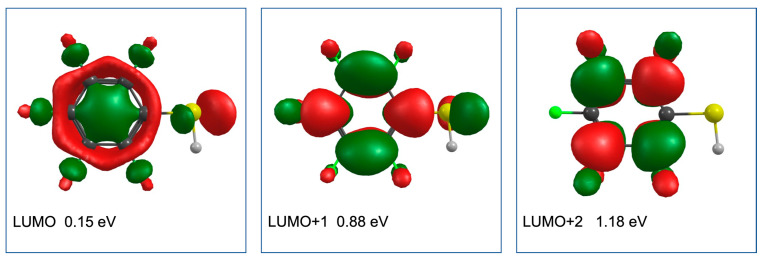
Contour plots of the LUMO, LUMO + 1, and LUMO + 2 of PFTP (B3LYP orbitals). The respective vertical electron attachment energy calculated using the EOM-EA-CCSD method with the B3LYP orbitals and aug-cc-pVTZ basis set are shown for each orbital. The LUMO has a σ* character, while LUMO + 1 and LUMO + 2 have a π* character and correlate with b_1_ (π*) and a_2_ (π*) MOs, respectively.

Figure 6 shows the LUMO, LUMO + 1, and LUMO + 2 of PFTP, along with the respective vertical electron attachment energies calculated using the EOM-EA-CCSD method with the B3LYP orbitals and aug-cc-pVTZ basis set. We note that their values are strongly dependent on the basis set while the order is reliable. Adhering to Jordan et al. [40], we labelled the π* orbitals according to the C_2V_ point group. The LUMO of PFTP was found to have an σ* character and is anti-bonding along the C–F and C–S coordinates, and there is a polarization along the S–H bond. From a hydrogen bonded S···H···F intermediate, this provides preferential conditions for HF loss from PFTP and the formation of [M-HF]^−^. This is consistent with the high efficiency of the [M-HF]^−^ formation at approximately 0 eV, assuming that it will proceed from the σ* electronic ground state of the anion. The vertical attachment energy to this state was found to be 0.15 eV. The LUMO + 1 of PFTP has a π* character and correlates with the b_1_ (π*) MO. The vertical attachment energy to this orbital is approximately 0.73 eV greater than the respective vertical attachment energy to the σ* electronic ground state of the anion. This may be explained by the strong inductive effect through the perfluorination stabilizing the σ* significantly stronger than the π*. Additionally, the mesomeric effect of the S atom is not strong enough to destabilize the b_1_ (π*) MO and push it above the a_2_ (π*) MO. This is due to the poor overlap of the 3p_x_ orbital of the S atom with the b_1_ (π) and b_1_ (π*) orbitals of the benzene ring. We attribute the asymmetry of the low energy peak in the [M-HF]^−^ ion yield from PFTP to dissociation through single electron occupation of π* LUMO + 1. The significantly higher intensity through the σ* ground state may in part be due to the direct dissociation from the σ* state, as compared to the required coupling of the π* with the σ* coordinate, even though such coupling should be promoted by the pJT, causing π*–σ* mixing through the out of plain bending of the fluorine and –SH substituents, as shown in Figure 4. However, the energy dependency of the autodetachment lifetime will also play a significant role. In fact, these effects are intertwined as the DEA cross-section is defined as the product of the electron-attachment cross-section and the survival probability of the TNI [51,52]. With less coupling and increased energy, the autodetachment process becomes more significant, reducing the survival probability with respect to dissociation, which in turn is reflected in lower DEA cross-sections at higher energies. This affects the shape of the peak in the ion yield curve, which appears asymmetric with a long tail on the right side.

**Figure 7:**
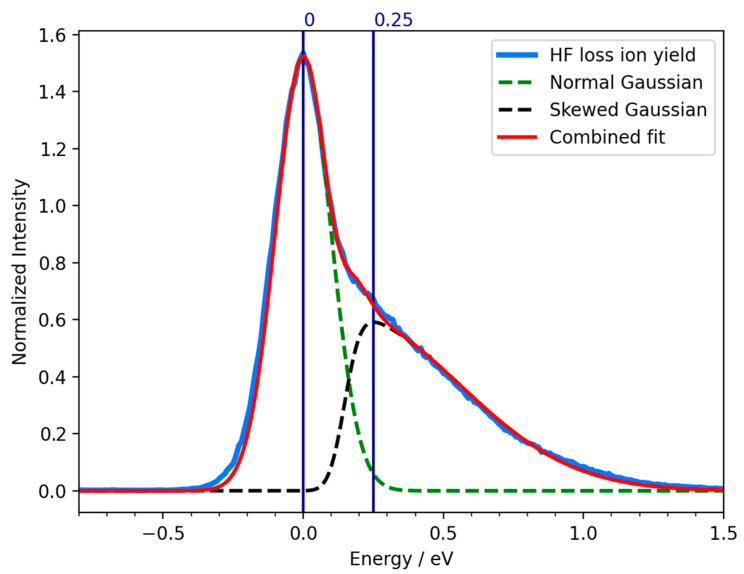
Combined fit of a Normal Gaussian (green dashed line) and a Skewed Gaussian (black dashed line) of the negative ion yield curve (blue line) for neutral HF loss and formation of [M-HF]^−^ upon DEA from PFTP. The resulting fit is represented by the red line.

This interpretation is demonstrated in Figure 7, where we present a fitting of the low energy contribution in the negative ion yield curve for neutral HF loss from PFTP upon DEA using a combined fit of normal and skewed gaussian curves. The fitting has been carried out with a python script using the LMFIT library [53]. For the lower energy contribution, the energy dependence of the autodetachment lifetime is neglected (hence, the normal Gaussian) and the natural width of the underlying resonance is considered to be well below the instrumental energy resolution. The FWHM of this contribution in the ion yield should thus reflects the energy resolution of the instrument, but in praxis it is approximately 250 meV. The skewed gaussian curve is chosen for the higher energy component to take into account the asymmetry of the peaks due to the energy dependence of the attachment process and the autodetachment lifetime [16]. With this approach, where we consider contributions from both the singly occupied LUMO and LUMO + 1, an excellent fit to the low-energy contribution in the [M-HF]^−^ ion yield from PFTP is obtained. 

This is consistent with the picture in the first low-energy resonance that appears with a peak intensity at 0 eV in the ion yield curves; the unpaired electron is temporarily accommodated in the σ* MO. In the second resonance, with a maximum contribution at 0.25 eV in the ion yield curve, the extra electron is temporarily placed in the b_1_(π^∗^) MO. Here, the autodetachment is significant, and the long tail on the right side of this contribution reflects the lower survival probability at higher attachment energies, due to the shorter lifetime of the respective temporary anion state. 

With respect to the LUMO + 2, shown in Figure 5, this correlates with the a_2_ (π*) MO. This A_2_ state has no electron density on the SH substituent, and a S···H···F hydrogen bond formation from this state is not to be expected. Correspondingly we do not expect a contribution from the A_2_ TNI to the [M-HF]^−^ formation. Furthermore, we expect both the [M-H]^−^ and [M-SH]^−^ formations to be direct channels that compete with the [M-HF]^−^ formation. These channels are slightly endothermic, as discussed above, and thus comparatively more efficient at higher energies. The ion yields for these fragments are correspondingly expected to derive their intensity from the high energy side of the σ* resonance and the b_1_(π*) resonance, either directly or through vibrational energy redistribution. Finally, the high-energy contribution at approximately 4.5–4.7 eV in the [M-HF]^−^, [M-H]^−^, and [M-SH]^−^ ion yield curves are most likely routed from the same resonance(s). 

**Figure 8:**
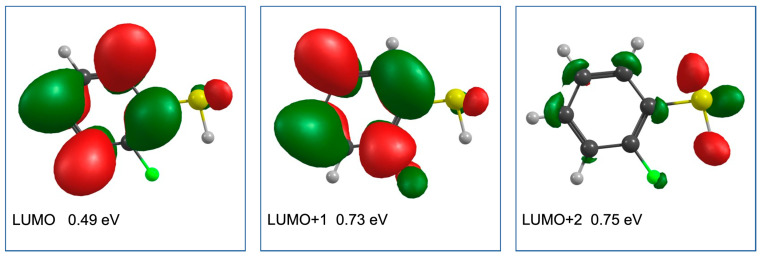
Contour plots of the LUMO, LUMO + 1, and LUMO + 2 of PFTP (B3LYP orbitals). The respective vertical electron attachment energy calculated using the EOM-EA-CCSD method with the B3LYP orbitals and aug-cc-pVTZ basis set are shown for each orbital. The LUMO and LUMO + 1 have a π* character and correlate with a_2_ (π*) and b_1_ (π*) MOs, respectively, while LUMO + 2 is a partial diffuse orbital with some electron density on the S and H atoms.

Figure 8 shows the LUMO, LUMO + 1, and LUMO + 2 of the 2-FTP, along with the respective vertical attachment energies calculated using the EOM-EA-CCSD method with the B3LYP orbitals and the aug-cc-pVTZ basis set. Different from PFTP, both the LUMO and LUMO + 1 in 2-FTP have a π* character and, in analogy to the nomenclature used for PFTP, they correlate with the a_2_(π*) and b_1_(π*) MOs, respectively. From these, the LUMO + 1 is anti-bonding along the C–F coordinate, providing a favorable condition for HF loss and the formation of [M-HF]^−^. However, different from the direct HF formation from the σ* SOMO in PFTP, this process is symmetry forbidden from the π* LUMO + 1 of 2-FTP and requires effective π*–σ*coupling. Calculated at the B3LYP D3BJ/aug-cc-pVTZ level of theory, we find the threshold for this process to be 0.42 eV, and we anticipate that the low relative cross section for the HF formation from 2-FTP is due to inefficient coupling of the LUMO + 1 with the respective C–F σ* state, in combination with the high threshold for this process. Hence, at these energies, autodetachment, and conceivably S^−^ formation, prevail over the HF formation. 

**Figure 9:**
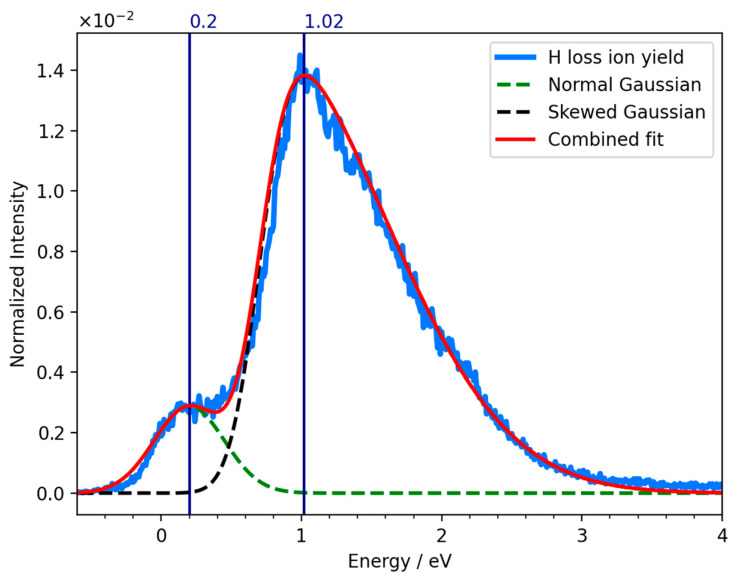
Single fit of a Skewed Gaussian (black dashed line) of the negative ion yield curve (blue line) for the H loss upon DEA from 2-FTP. Additionally, a Normal Gaussian peaking at 0.2 eV (green dashed line) is included to reproduce the impurity contribution at 0 eV. The red line represents the resulting fit.

The hydrogen loss from 2-FTP is by far the most efficient DEA channel for this molecule and is characterized in the ion yields by a broad asymmetric contribution peaking at 0.88 eV and tailing off towards higher energies. A shoulder at approximately 0 eV in the ion yield curve is also observed, which we attribute to ‘hot -band transitions’ or I^−^ (*m*/*z* = 127) formation from iodine containing contaminations. The thermochemical threshold for the hydrogen loss, calculated at the B3LYP D3BJ/aug-cc-pVTZ level of theory, is found to be 0.82 eV, and we anticipate that this process proceeds predominantly from the partial diffuse LUMO + 2 orbital, which has some electron density on the S and H atoms. This assignment is also supported by the single contribution fit to the ion yield curve shown in Figure 9, where an excellent agreement is obtained by a fit of a single skewed Gaussian to the hydrogen loss ion yield. For completeness, a Gaussian contribution peaking at 0.2 eV is also included to reproduce the 0 eV impurity contribution. In principle, all conditions for HF formation could also proceed from the LUMO + 2; however, at these energies the hydrogen-bonded intermediate is not stable and the direct hydrogen loss prevails as the most efficient channel. 

In a sense, DEA can be compared to photo dissociation as both are effectuated by a single electron occupation of previously unoccupied antibonding orbitals. In this context, we note a recent study by Marchetti et al. [34] on near ultraviolet spectroscopy and the photodissociation dynamics of 2- and 3-substituted thiophenols. There it was shown for 2-FTP that the repulsive S–H ^1^nσ* state crosses the ^1^ππ* state close to its vibrational ground state. Population transfer from the π* to the repulsive S–H σ* may thus proceed through non-adiabatic coupling above the respective vibrational ground state, but tunnelling would be required from the ground state. In the current terminology, this may offer an alternative path for HF formation from the π* LUMO of the TNI formed in the initial attachment process.

It is clear from the current experiments and calculations that the perfluorination in PFTP, as compared to 2-FTP, does not only lower the thermochemical threshold for the HF loss in DEA, but also lowers the lowest σ* MO below the respective π* MOs, providing a very favorable condition for the HF loss. Potentially, this may be taken advantage of to promote the interaction of radiosensitizers with low-energy electrons, thus increasing their efficiency.

## 3. Materials and Methods

### 3.1. Experimental Setup

Negative ion yield curves were recorded by means of a crossed electron-molecular beam apparatus. The experimental setup has been described in detail previously [54] and only a short description will be given here. The instrument is composed of a trochoidal electron monochromator (TEM), an effusive gas inlet system, and a quadrupole mass spectrometer (Hiden EPIC1000). The monochromator was heated to 120 °C with two internal halogen lamps to avoid condensation of the target gas on the electrical lens components. A quasi mono-energetic electron beam, generated with the TEM, crosses a molecular beam of the target gas obtained through the effusive gas inlet system. The ions resulting from the electron-molecule interactions in the collision region are then extracted through a small electric field (<1 V/cm) and analyzed by mass spectrometer. The DEA ion yield curves are recorded by scanning through the relevant electron energy at a fixed mass (*m*/*z*). The compounds were purchased from ABCR GMBH & Co. (Karlsruher, Germany), with a stated purity of 97% for PFTP and 98% for 2-FTP.

The electron energy was calibrated to the well-known 0 eV resonance for SF_6_^−^ formation from SF_6_, and the energy resolution of the electron beam at 0 eV was determined from the FWHM and was in the range of 120–140 meV. The background pressure inside the chamber was approximately 3 × 10^8^ mbar, while the sample gas pressure was in the range of (2–10) × 10^7^ mbar for PFTP and (1–4) × 10^6^ mbar for 2-FTP. 

### 3.2. Theoretical Procedures

All quantum chemical calculations were carried out using ORCA version 4.2.1 [55].

Geometry optimization of all the charged fragments and neutral molecule were performed at the B3LYP [56,57,58] level of theory, using the aug-cc-pVTZ basis set [59,60] and D3(BJ) dispersion correction [61,62]. For the closed shell systems, the Restricted Kohn–Sham (RKS) formalism was used, while the Unrestricted Kohn–Sham (RKS) formalism was used for open-shell systems. Harmonic vibrational frequencies were calculated at the same level of theory to derive zero-point vibrational energy (ZPVE) and thermal energy corrections. All threshold calculations refer to the single point energy of the relaxed structures, and they have been performed at the same level of theory as the geometry optimization. The reaction enthalpies at 0K (ΔH_0K_) were calculated by subtracting the total energy of all fragments from the total energy of the parent molecule, including the respective ZPVEs. The thermally corrected thresholds (E_th_) were obtained by subtracting the thermal energy of the parent molecule at room temperature from the reaction enthalpies at 0K. 

In addition, for HF formation from PFP and PFTP, geometry optimizations and harmonic vibrational frequencies calculation were also performed at the ⍵B97X-D3 [61,63]/aug-cc-pVTZ level of theory, and single-point energy calculations were performed at the wB97X-D3/aug-cc-pVQZ [59,60] and DLPNO-CCSD(T) [64,65,66,67]/aug-cc-pVQZ level on the respective ωB97X-D3 optimized geometries. In the DLPNO-CCSD(T) calculations, for open shell systems, quasi-restricted orbitals (QROs) [68] were used as a reference determinant from the UHF orbitals. ZPVEs and thermal energy correction for the neutral were obtained from the ωB97X-D3 vibrational frequencies calculation. These calculations are given as Supplementary Information in Table S1.

Vertical electron attachment energies to the virtual orbitals of PFTP and 2-FTP were calculated using the EOM-EA-CCSD method with B3LYP orbitals and aug-cc-pVTZ basis set.

The minimum energy path for the HF formation and rearrangement mechanism was calculated using the Nudged Elastic Band method with transition state (TS) optimization (NEB-TS) [69] at the B3LYP D3BJ level, using the aug-cc-pVTZ basis set. For the reactants and products, we used the optimized geometry at the B3LYP D3BJ/aug-cc-pVTZ level of theory. The transition states were checked through the calculation of harmonic vibrational frequencies, and confirmed as effective saddle points, and it was found that they had only one negative frequency (imaginary mode).

Generally, basis set superposition errors (BSSE) are small in DFT as compared to methods based on wave-function theory (WFT), due to the lower basis set sensitivity. With respect to the current study, such errors are well within the experimental energy resolution and are not taken into account.

Finally, neither spin orbit coupling nor relativistic effects were taken into account. These effects are small for the current systems and well within the limit of our experimental accuracy.

## 4. Conclusions

Here we presented a combined theoretical and experimental study on DEA in relation to PFTP and 2-FTP, exploring the influence of perfluorination on the susceptibility of these compounds to DEA. We reported the energy dependence of the relative DEA cross-sections for the observed fragments and the thermochemical thresholds calculated for the respective DEA processes, as well as the reaction paths computed for the formation of neutral HF up on DEA to these compounds. We also showed the nature of the LUMOs involved in the initial electron attachment process and the respective transitions energies. 

We found that the perfluorination in PFTP, as compared to 2-FTP, influences significantly the DEA processes. While the dominant DEA channel in PFTP is the HF loss, direct H loss is the dominating DEA process in 2-FTP, and HF loss is insignificant. We attribute this to the exothermic nature of the HF formation from PFTP, provided not only by the energy gain through the HF formation, but also by the perfluorination. Hence, the perfluorination increases the electron affinity of the charge-retaining fragment, providing additional energy in the process. This is reflected in the respective ion yield curve for the HF loss from PFTP, which is characterized by high relative cross sections already at the 0 eV threshold. In fact, it is 5 orders of magnitude higher than the relative cross section for HF formation from 2-FTP. The key point here is that, due to the very high electron attachment cross sections at very low energies, DEA may be made very efficient by tailoring exothermic reaction channels into the respective molecules. The perfluorination also influences the molecular orbital structure, and specifically the energy ordering of the lowest lying MOs. This is due to the dominating inductive effect of fluorine providing significantly stronger stabilization for the lowest lying σ* orbitals than the respective π* orbitals. This is very important with respect to the DEA efficiency as effective coupling of the lowest lying orbitals with the respective dissociation coordinates is essential for DEA to be effective at low electron attachment energies. For PFTP, this is provided because the lowest lying virtual orbital is of a σ* character, it is anti-bonding along the C–F and C–S coordinates and is polarized along the S–H bond. Hence, single electron occupation of this orbital provides all prerequisites for “direct” HF formation at very low attachment energies. The two lowest lying virtual MOs of 2-FTP, on the other hand, are antibonding π^∗^ orbitals. Different from the direct HF formation from single electron occupation of the σ* LUMO in PFTP, this process is symmetrically forbidden from these π^∗^ orbitals and requires effective π*–σ*coupling to proceed. Aided by the slight endothermicity, this puts relaxation through dissociation at a disadvantage compared to relaxation through re-emission of the electron, rendering the low energy DEA processes for 2-FTP inefficient as compared to PFTP.

It is clear from the current study, as well as from many previous DEA studies in the literature, that perfluorination enhances the susceptibility of many compounds towards low energy electrons, and rearrangement reactions such as HF formation may be used to open up exothermic DEA channels. In the context of the role of DEA in the functionality of radio sensitizers, these may be seen as important tools to promote efficient DEA reactions at low electron energies, and we argue that such tools may be valuable for a bottom-up approach in the design of efficient radiosensitizers.

## Figures and Tables

**Table 1:** Calculated 0K reaction enthalpies (ΔH_0K_) and thermally corrected thresholds (ΔE_th_) for the fragments observed in DEA to PFTP and 2-FTP. For the HF loss, the values are shown for both the direct process, [M-HF]^−^/C_6_F_4_S^−^ or C_6_H_4_S^−^ and for the rearrangement process shown in Figure 2, [M-HF]^−^/C_5_F_4_CS^−^ or C_6_F_4_S^−^. At the bottom of the table, the same values are calculated for the HF formation upon DEA to PFP. The calculations are performed at the B3LYP D3BJ/aug-cc-pVTZ level of theory.

*m*/*z*	Fragment	ΔH_0K_	ΔE_th_
**PFTP**			
199	[M-H]^−^/C_6_F_5_S^−^	0.42	0.13
180	[M-HF]^−^/C_6_F_4_S^−^	−0.0084	−0.29
180	* [M-HF]^−^/C_5_F_4_CS^−^	−0.066	−0.35
167	[M-SH]^−^/C_6_F_5_^−^	0.61	0.32
**2-FTP**			
127	[M-H]^−^/C_6_H_4_FS^−^	1.03	0.84
108	[M-HF]^−^/C_6_H_4_S^−^	0.62	0.42
108	* [M-HF]^−^/C_5_H_4_CS^−^	0.47	0.28
32	S^−^	0.33	0.14
**PFP**			
164	[M-HF]^−^/C_6_F_4_O^−^	−0.066	−0.33
164	* [M-HF]^−^/C_5_F_4_CO^−^	−0.27	−0.54

* Calculated threshold considering the rearrangement of the ring after the HF formation.

## Data Availability

The data underlying this article will be shared on reasonable request from the corresponding author.

## Supplementary Materials

The following supporting information can be downloaded at: https://www.mdpi.com/article/10.3390/ijms23052430/s1.
