# Supplementary material for: HF Formation through Dissociative Electron Attachment—A Combined Experimental and Theoretical Study on Pentafluorothiophenol and 2-Fluorothiophenol
Source: Int J Mol Sci. 2022 Feb 23;23(5):2430. doi: 10.3390/ijms23052430 (PMC8910151; doi:10.3390/ijms23052430)
Supplement: Supplementary file 1 [file ijms-23-02430-s001.zip › ijms-1585340-supplementary.pdf]

Supplementary Information

Table S1. Calculated thermally corrected thresholds of HF formation upon DEA to PFP and PFTP. The calculations are performed at the ωB97X-D3/ aug-cc-Pvtz, ωB97X-D3/ aug-cc-pVQZ and DLPNO-CCSD (T) aug-cc-pVQZ levels of theory. Geometry optimization was carried out at the wB97X-D3/ aug-cc-pVTZ. ZPVEs and thermal energy correction for the parent neutral molecule were obtained from the frequencies calculation performed at the same level of theory that was used for the optimization.

Fragment	ωB97X-D3/ aug-cc-pVTZ	ωB97X-D3/ aug-cc-pVQZ	DLPNO-CCSD(T)/ aug-cc-pVQZ
PFP			
$[M - HF]^-/C_5F_4CO^-$ *	–0.45	–0.45	–0.50
$[M - HF]^-/C_6F_4O^-$	–0.047	–0.050	–0.18
PFTP			
$[M - HF]^-/C_5F_4CS^-$ *	–0.21	–0.23	–0.35
$[M - HF]^-/C_6F_4S^-$	–0.16	–0.16	–0.34

* Calculated threshold considering the rearrangement of the ring after the HF formation
